# Lipome géant pelvien mimant un liposarcome

**DOI:** 10.11604/pamj.2019.33.20.15008

**Published:** 2019-05-14

**Authors:** Bacha Dhouha, Walha Maroua, Baccouch Seifeddine, Talbi Ghofrane, Gharbi Lassaad, Bayar Rached, Mzabi Rgaya

**Affiliations:** 1 Université de Tunis El Manar, Faculté de Médecine de Tunis, Service d'Anatomie Pathologique, Hôpital Mongi Slim, Sidi Daoued, Tunis; 2 Université de Tunis El Manar, Faculté de Médecine de Tunis, Service de Chirurgie Viscérale, Hôpital Mongi Slim, Sidi Daoued, Tunis

**Keywords:** Lipome géant, liposarcome, pelvis, Giant lipoma, liposarcoma, pelvis

## Abstract

Le lipome géant (LG) est une entité rare, de siège ubiquitaire. Sa localisation pelvienne est rare avec moins de 10 cas rapportés dans la littérature. Le principal diagnostic différentiel est le liposarcome bien différencié de type lipoma like. Nous rapportons le cas d'une patiente âgée de 50 ans, qui avait consulté pour une gêne pelvienne depuis 3 mois avec l'apparition d'une masse ischio rectale gauche. Le scanner abdomino-pelvien avait montré une masse lipomateuse homogène hypo dense en situation pré sacrée de 10 x18 cm. A l'imagerie par résonance magnétique (IRM), cette masse était hyper intense en pondération T1 et T2 avec des cloisons fines et atteignait la hauteur de la 2^ème^ vertèbre sacrée. Une exérèse totale a été réalisée par une double voie abdominale et périnéale sans rupture de sa capsule. L'examen anatomopathologique a confirmé le diagnostic de LG. Le but de ce travail était de rapporter un nouveau cas de LG pelvien pré sacré qui se prolonge dans la fosse ischio rectale gauche.

## Abstract (en)

Giant lipoma (GL) is a rare ubiquitous tumor. Pelvic lipoma is rare with less than 10 cases reported in the literature. The main differential diagnosis is lipoma-like well differentiated liposarcoma. We report the case of a 50-year old female patient, with a 3-month history of pelvic discomfort, presenting with a mass in the left ischiorectal fossa. Abdominopelvic CT scan showed presacral hypodense homogeneous lipomatous mass measuring 10x18 cm. MRI showed hyperintense lesion on T1 and T2 -weighted images with fine partition walls reaching the 2^nd^ sacral vertebra. Total abdominoperineal resection was performed without rupture of tumor capsule. Anatomo-pathological examination confirmed the diagnosis of GL. This study aims to report a new case of giant presacral pelvic lipoma which extends into the left ischiorectal fossa.

## Introduction

Le lipome est une tumeur bénigne encapsulée, caractérisée par la prolifération d'adipocytes matures, sans atypies cyto-nuclaires [1, 2]. Il est appelé géant lorsque son diamètre dépasse 10 cm [2]. Il s'agit d'une entité rare, de siège ubiquitaire. Le principal diagnostic différentiel est le liposarcome bien différencié de type lipoma like [1, 3]. Le but de ce travail est de rapporter un nouveau cas de lipome géant (LG) pelvien pré sacré qui se prolonge dans la fosse ischio rectale gauche.

## Patient et observation

**Figure 1:**
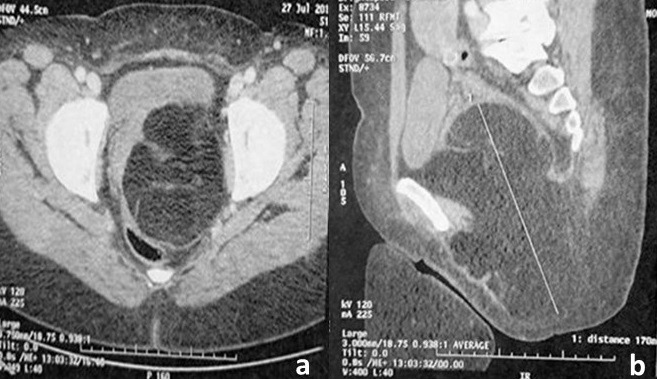
TDM abdomino-pelvienne: (A) coupe transversale d’une volumineuse masse lipomateuse pré sacrée, homogène et hypo dense; (B) elle se prolonge dans la fosse ischio rectale gauche à travers le périnée: coupe sagittale

**Figure 2:**
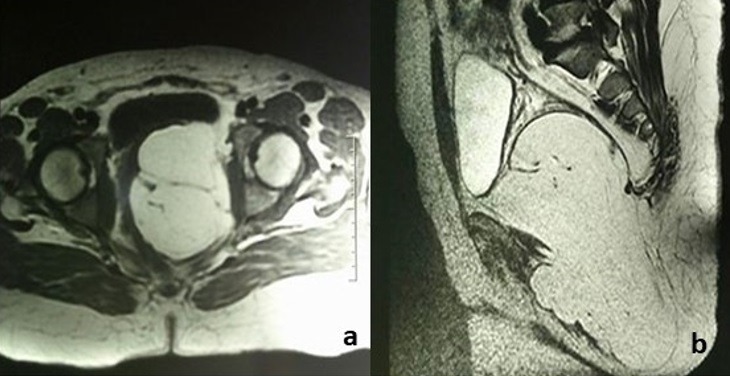
IRM abdomino-pelvienne: (A) coupe transversale d’une masse hyper intense en pondération T1 avec des cloisons fines; (B) elle atteint la hauteur de la 2^ème^ vertèbre sacrée: coupe sagittale

**Figure 3:**
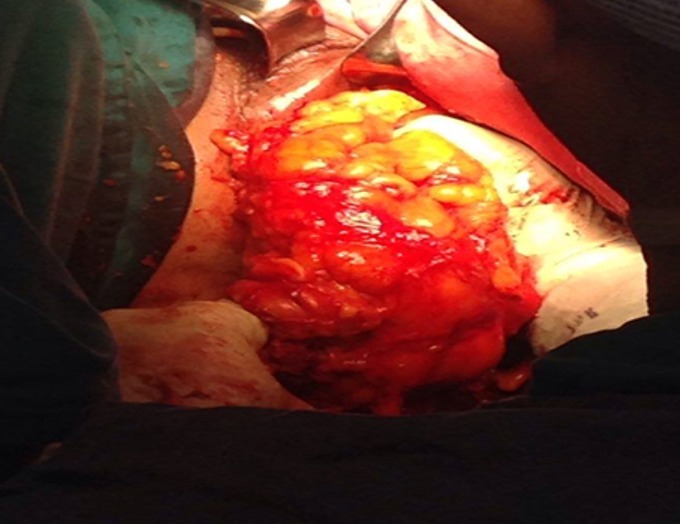
extraction peropératoire par voie périnéale d’une volumineuse masse graisseuse

**Figure 4:**
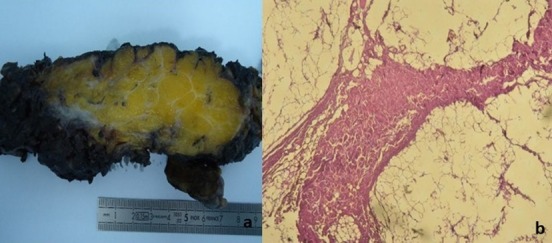
(A) aspect jaunâtre de la tumeur avec des septa fibreux à la coupe; (B) prolifération d’adipocytes matures sans atypies cellulaires: Hématoxyline Eosine x 40

Une patiente âgée de 50 ans avait consulté pour une gêne pelvienne depuis 3 mois avec l'apparition depuis 1 mois d'une masse ischio rectale gauche « molasse ». Son état général était conservé et l'examen abdomino-pelvien était sans particularités. Le scanner abdomino-pelvien avait montré une volumineuse masse lipomateuse homogène hypodense, de siège pré sacré, mesurant 10 x 18 cm (Figure 1A). Cette masse se prolongeait dans la fosse ischio rectale gauche à travers le périnée (Figure 1B). A l'IRM, la masse présentait des cloisons fines et était hyper intense en pondération T1 et T2 (Figure 2A). Sur les coupes sagittales, elle atteignait la hauteur de la 2^ème^ vertèbre sacrée (S2) (Figure 2B). Le diagnostic pré-opératoire était soit celui d'un liposarcome pelvien ou d'un LG. Une exérèse totale a été réalisée par une double voie abdominale et périnéale sans rupture de la capsule tumorale (Figure 3). Les suites opératoires étaient simples. L'examen anatomopathologique trouvait une masse de 800 grammes, jaunâtre avec quelques fins septa périphériques (Figure 4A). Il s'agissait d'une prolifération d'adipocytes matures sans atypies ni lipoblastes (Figure 4B). Il n'existait pas d'amplification du gène MDM2 en étude cytogénétique. Le diagnostic de LG était confirmé. L'évolution était favorable, sans signe de récidive après un recul de 18 mois.

## Discussion

Le LG est une tumeur adipocytaire bénigne. Il est de siège ubiquitaire mais touche le plus souvent le tronc et les membres. Au niveau tronculaire, il peut être pariétal ou profond, rétro péritonéal ou pelvien [2]. Le LG pelvien est rare avec moins de 10 cas rapportés dans la littérature. Il touche les sujets adultes entre 40 et 60 ans, sans prédominance de sexe [2, 4]. Le LG pelvien est habituellement pré sacré avec parfois des prolongements vers la région inguinale ou dans le périnée à travers le trou sciatique, le trou obturateur ou le plancher pelvien, comme dans notre cas [2]. Dans ces cas, le contingent profond pelvien est beaucoup plus volumineux que celui superficiel, qui est palpable, réalisant un aspect en « iceberg ». Les signes cliniques sont peu spécifiques à type de gêne pelvienne. Des signes de compression peuvent s'observer. Il peut s'agir d'une compression de la charnière de la veine fémorale, en cas de prolongement inguinal, ou des sciatalgies, en cas de prolongement dans le trou sciatique [1-3,5]. La palpation d'une masse « molasse » simulant une hernie peut être un signe de découverte de la tumeur. Les explorations radiologiques permettent d'évoquer le diagnostic de LG [1, 2]. A l'échographie, il est d'échogénicité variable, homogène et avasculaire au temps doppler. Au scanner, la masse est homogène et hypodense, sans prise de contraste. Des prolongements du LG vers la région inguinale ou dans le périnée doivent être recherchés. Le niveau de la tumeur par rapport au bord de la 3^ème^ vertèbre sacrée doit être précisé pour la prise en charge thérapeutique. L'IRM est l'examen de référence pour l'exploration du LG. Il parait hyper intense en pondération T1 et T2, homogène, s'effaçant après saturation de la graisse et ne se rehausse pas après injection intraveineuse de Gadolinium [1]. Les septa, lorsqu'ils existent, sont toujours fins (< 2mm) [1]. En cas de doute diagnostique, en particulier avec le liposarcome, une biopsie doit être envisagée [2]. Le traitement chirurgical consiste en une exérèse complète du LG sans rupture de sa capsule. Lorsque la tumeur dépasse la hauteur de la 3^ème^ vertèbre sacrée, une double voie d'abord abdominale, par laparotomie ou laparoscopie, et périnéale s'impose [6, 7]. Dans le cas contraire, la voie périnéale suffit. Dans notre cas la voie d'abord était double abdominale et périnéale car la masse atteignait la hauteur de la 2^ème^ vertèbre sacrée. Le diagnostic de certitude est anatomo-pathologique. L'examen histologique trouve une prolifération d'adipocytes matures sans atypies cytologique ni lipoblastes. L'immunohistochimie montre l'absence de marquage des cellules tumorales par les anticorps anti MDM2 et/ou anti CDK4 et la biologie moléculaire montre l'absence d'amplification des gènes correspondants [8]. Les récidives sont possibles dans 2 à 5% des cas, en particulier en cas de résection incomplète [2]. La surveillance doit ainsi être régulière et prolongée avec une IRM annuelle et en cas du moindre symptôme. Les résections répétées favoriseraient la transformation du lipome en liposarcome [6].

## Conclusion

Le LG pelvien est une entité rare. L'IRM est l'examen de choix pour évoquer le diagnostic et planifier la stratégie thérapeutique. Le diagnostic est confirmé à l'histologie, aidée par l'immunohistochimie et l'étude par biologie moléculaire à la recherche d'une amplification des gènes MDM2 et/ou CDK4. L'exérèse complète sans rupture de la capsule tumorale est le traitement de choix par voie périnéale et/ou abdominale. La surveillance doit être régulière et prolongée.

## Conflits d’intérêts

Les auteurs ne déclarent aucun conflit d'intérêts.
